# A J-shaped link between body roundness index and albuminuria in U.S. adults: insights from NHANES 2005–2020

**DOI:** 10.1080/0886022X.2025.2567035

**Published:** 2025-10-08

**Authors:** Xiang Lei, Xiaotong Peng, Xue Liu, Yuanyuan Li

**Affiliations:** a Department of Pediatrics, the Central Hospital of Shaoyang, Shaoyang, Hunan, China; b Department of Emergency Medicine, Yueyang Central Hospital, Yueyang, Hunan, China; c Department of Emergency Medicine, The Affiliated Changsha Central Hospital, Hengyang Medical School, University of South China, Changsha, Hunan, China

**Keywords:** Body roundness index, albuminuria, obesity-related metrics, NHANES, abdominal obesity

## Abstract

**Background:**

The relationship between Body Roundness Index (BRI) and albuminuria remains uncertain. This study examined the association between BRI, an innovative anthropometric metric, and albuminuria in a representative sample of adults.

**Methods:**

This study used data from the National Health and Nutrition Examination Survey (NHANES) spanning 2005 to 2020. BRI values were computed for 37,826 eligible participants. Urine albumin-to-creatinine ratio (ACR) values equal to or exceeding 30 mg/g were classified as albuminuria. Statistical analyses included weighted multivariable logistic regression models, two-piecewise linear regression models, smooth curve fittings, subgroup analyses, and interaction assessments.

**Results:**

In the fully adjusted model, the odds ratio (OR) for the relationship between BRI and albuminuria was 1.18 (95% CI: 1.12–1.24). Furthermore, a non-linear J-shaped relationship was observed with a turning point at a BRI value of 3.08. Subgroup analyses revealed a stronger association in males (OR: 1.31, 95% CI: 1.24–1.39) than in females (OR: 1.11, 95% CI: 1.05–1.18).

**Conclusion:**

Our study identified a J-shaped relationship between BRI and albuminuria in adults.

## Introduction

Albuminuria is defined by the presence of albumin in the urine, with an albumin-to-creatinine ratio (ACR) of ≥30 mg/g indicating its occurrence [1]. The condition reflects increased permeability of the glomerular filtration barrier, commonly caused by systemic factors, including diabetes mellitus and hypertension, or by renal-specific diseases [2,3]. Albuminuria is widely acknowledged as a critical sign of deteriorating renal function in patients with obesity-related nephropathy and diabetic nephropathy [4]. Prior studies have consistently identified albuminuria as a major determinant of cardiovascular outcomes, regardless of conventional risk factors [5–8]. Early detection and management of albuminuria are crucial in slowing disease progression and improving clinical outcomes.

Obesity is a global health crisis. According to the World Obesity Atlas 2024, obesity prevalence is steadily increasing, with projections indicating that nearly 3.3 billion adults will have a high BMI (overweight or obese) by 2035 [9]. Beyond general obesity, central obesity, marked by visceral fat accumulation, is particularly concerning due to its higher metabolic risks compared to peripheral obesity [10]. BMI is frequently utilized to assess obesity due to its simplicity and practicality. However, it has no limitations, particularly its inability to differentiate between fat types or accurately estimate visceral fat accumulation [11]. To address these shortcomings, BRI was developed as a new body measurement index combining waist circumference and height. By reflecting fat distribution and visceral adiposity more accurately, BRI has demonstrated superiority over BMI in predicting risks associated with cardiovascular disease and metabolic syndrome [12]. Research on BRI’s clinical utility is emerging. Studies have shown its potential in identifying obesity-related health risks, including cardiovascular disease [13–15]. Notably, Xuankai Qin et al. identified a reverse association between BRI and albuminuria in non-adults, indicating a unique relationship within younger age groups [16]. However, whether these findings extend to adult populations remains unclear.

As of now, no study has examined the relationship between BRI and albuminuria in adults. This research seeks to fill this gap by exploring the relationship between BRI, a new body measurement index reflecting body shape and fat distribution, and albuminuria, an established marker of kidney dysfunction and cardiovascular risk. Understanding this relationship may offer important perspectives on the clinical value of BRI as a screening tool for early kidney damage and its potential role in guiding interventions to reduce associated health risks.

## Materials and methods

### Data source and population study

This study used data from NHANES (2005–2020). NHANES adopts a stratified, multistage sampling method to ensure representation of diverse population groups. Participants provided demographic, clinical, and laboratory data, with ethical approval and informed consent ensured. Detailed methodologies and datasets are publicly accessible through the NHANES website.

**Figure 1.**
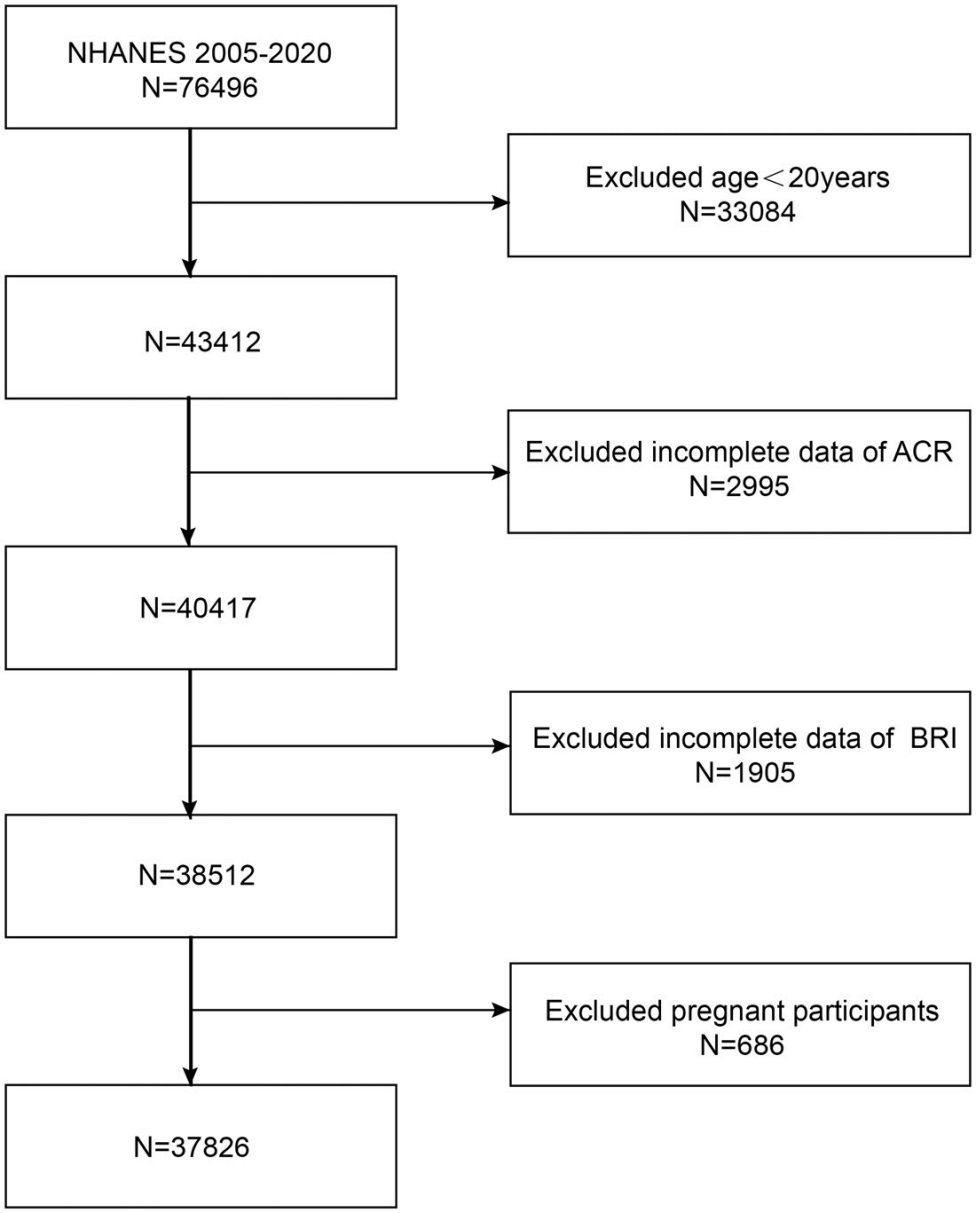
**Flowchart of the sample selection from NHANES 2005–2020.** The initial sample consisted of 76,496 participants. After excluding individuals under 20 years of age (*N* = 33,084), those with missing data on ACR (*N* = 2,995) or BRI (*N* = 1,905), and pregnant participants (*N* = 686), the final dataset included 37,826 eligible adults.

The initial sample consisted of 76,496 participants. After excluding individuals under 20 years of age (*N* = 33,084), those with missing data on ACR (*N* = 2,995) or BRI (*N* = 1,905), and pregnant participants (*N* = 686), the final dataset included 37,826 eligible adults (Figure 1). The age threshold of 20 years was selected because NHANES defines individuals aged ≥20 years as adults.

### Definition of BRI

BRI values were calculated using the following formula: $\text{BRI}=364.2-365.5\times \sqrt{1-\frac{{(\text{waist circumference}/2\pi )}^{2}}{{\left(0.5\times \text{height}\right)}^{2}}}$ [12]. Higher BRI values indicate individuals with a more rounded body shape, while values closer to 1 are typically associated with leaner individuals with a narrower body structure. In this investigation, BRI was treated as the independent variable. To facilitate comparison and detect non-linear trends, we categorized BRI into tertiles based on its distribution in the study sample. This data-driven approach is commonly used in epidemiological studies where no established clinical cutoffs exist. The tertile cutoff points were as follows: Tertile 1: 1.05–4.30, Tertile 2: 4.30–6.12, Tertile 3: 6.12–22.41.

### Determination of albuminuria

Random urine samples collected at the MEC were analyzed for albumin and creatinine concentrations. Albuminuria was identified based on an ACR of ≥30 mg/g, calculated by dividing albumin concentration (mg/L) by creatinine concentration (g/L) [17,18]. In this investigation, albuminuria was considered the dependent variable.

### Covariates

The analysis adjusted for a comprehensive set of covariates. Demographic variables included age, sex, and race/ethnicity. Behavioral factors encompassed smoking status, drinking status, and physical activity levels. Socioeconomic indicators, such as education level and poverty-income ratio (PIR), were also factored in. Clinical variables comprised BMI, diabetes and hypertension status, dietary factors (sodium and protein intake), liver function markers (ALT and AST), lipid profile (total cholesterol and triglycerides), and renal function markers (serum creatinine); serum uric acid was included as a metabolic variable due to its relevance to obesity and cardiovascular health. Sodium and protein intake data were calculated as the average of Day 1 and Day 2 total nutrient intakes, derived from two 24-h dietary recall interviews conducted by NHANES. These values reflect participants’ reported intake over two nonconsecutive days and are commonly used in dietary analyses to reduce day-to-day variation. These covariates were selected based on their established relevance to obesity and albuminuria, ensuring a strong adjustment for confounding influences.

### Statistical analysis

All data were analyzed using R software 4.2 and Empower Stats software 6.0. A *p*-value of <0.05 is considered statistically significant. Descriptive statistics, including means, standard errors, and percentages, were applied to outline the baseline characteristics of the participants. Weighted multivariable logistic regression models were applied, taking into account the sampling weights to ensure the results were representative of the U.S. adult population. These models were adjusted for potential confounders, including demographic factors, clinical variables, and health conditions. To compare the predictive value of BRI against conventional anthropometric measures, we repeated the main logistic regression analyses using BMI in place of BRI. To further explore the independent value of BRI, we included both BRI and BMI in the same multivariable logistic regression model. A two-piecewise linear regression model and smooth curve fitting were used to detect non-linear relationships and identify turning points. To handle missing covariate data and reduce potential bias, we performed multiple imputation using chained equations (MICE) with five imputations. The imputed datasets were pooled using Rubin’s rules, and results were compared with those from the complete-case analysis to assess consistency. Subgroup analyses were conducted to examine variations across different subgroups. Interaction effects were tested by including interaction terms (BRI × sex and BRI × age) in the fully adjusted multivariable logistic regression models to investigate whether the relationship between BRI and albuminuria differed by these characteristics. To account for multiple subgroup comparisons and reduce the risk of Type I error, the False Discovery Rate (FDR) correction method was applied to the p-values obtained from interaction terms.

## Results

### Baseline characteristics

This study included 37,826 participants, characterized by an average age of 47.16 ± 0.23 years. Among them, 51.05% were female and 48.95% were male. Participants were categorized into three BRI tertiles: Tertile 1 (1.05–4.30), Tertile 2 (4.30–6.12), and Tertile 3 (6.12–22.41). Compared to Tertile 1, Tertile 3 had a higher proportion of females, Mexican Americans, subjects with lower educational attainment, and participants with diabetes or hypertension. In terms of physical and laboratory characteristics, Tertile 3 exhibited elevated levels of BMI, ALT, triglycerides, and serum uric acid. Albuminuria was present in 9.15% of the participants overall, with its prevalence increasing across BRI tertiles (Tertile 1: 6.27%, Tertile 2: 8.21%, Tertile 3: 13.87%), as detailed in Table 1.

**Table 1.** Baseline characteristics of study population according to body roundness index tertiles.

		Tertile1 (mg/g)	Tertile2 (mg/g)	Tertile3 (mg/g)	
Body Roundness Index	Overall	(1.05–4.30)	(4.30–6.12)	(6.12–22.41)	*P* for trend
Sample size	*N* = 37826	*N* = 12609	*N* = 12608	*N* = 12609	
Age (year)	47.16 ± 0.23	41.32 ± 0.36	49.77 ± 0.18	51.64 ± 0.18	<0.0001
**Sex (% [SE])**					<0.0001
Male	48.95 (0.37)	50.36 (0.59)	54.58 (0.61)	40.82 (0.64)	
Female	51.05 (0.37)	49.64 (0.59)	45.42 (0.61)	59.18 (0.64)	
**Race (% [SE])**					<0.0001
Mexican American	8.42 (0.13)	5.65 (0.14)	10.07 (0.25)	10.08 (0.27)	
Other Hispanic	5.5 (0.11)	4.65 (0.17)	6.16 (0.2)	5.85 (0.19)	
Non-Hispanic White	67.68 (0.28)	68.9 (0.5)	67.25 (0.47)	66.61 (0.56)	
Non-Hispanic Black	11.06 (0.13)	10.91 (0.24)	9.52 (0.22)	13 (0.27)	
Other Races	7.33 (0.15)	9.9 (0.33)	7.01 (0.28)	4.45 (0.22)	
**Education level (% [SE])**					<0.0001
Less than high school	16.51 (0.46)	12.75 (0.35)	17.64 (0.39)	20.03 (0.5)	
High school or GED	22.67 (0.63)	19.61 (0.46)	23.33 (0.53)	25.81 (0.55)	
Above high school	60.77 (0.70)	67.56 (0.52)	59 (0.6)	54.14 (0.68)	
Others	0.05 (0.02)	0.08 (0.03)	0.04 (0.02)	0.03 (0.01)	
**Smoking status (% [SE])**					<0.0001
Never	54.65 (0.72)	57.17 (0.61)	53.55 (0.75)	52.68 (0.61)	
Former	24.54 (0.64)	18.84 (0.47)	26.72 (0.57)	29.34 (0.62)	
Current	20.76 (0.60)	23.96 (0.51)	19.69 (0.55)	17.91 (0.49)	
Unknown	0.05 (0.02)	0.04 (0.01)	0.04 (0.02)	0.07 (0.03)	
**Alcohol use (% [SE])**					<0.0001
Heavy alcohol consumption	55.14 (0.71)	59.88 (0.61)	54.3 (0.66)	50.08 (0.64)	
Moderate alcohol consumption	6.33 (0.34)	6.56 (0.29)	6.43 (0.3)	5.92 (0.3)	
Mild alcohol consumption	22.15 (0.58)	17.79 (0.49)	22.98 (0.49)	26.73 (0.54)	
Unknown	16.38 (0.53)	15.76 (0.43)	16.29 (0.46)	17.27 (0.44)	
**PIR (% [SE])**					<0.0001
≤1.3	19.84 (0.5)	18.36 (0.38)	18.59 (0.46)	23.13 (0.48)	
>1.3 and ≤3.5	33.3 (0.70)	31.16 (0.53)	32.91 (0.61)	36.45 (0.58)	
>3.5	40.26 (0.75)	44.22 (0.58)	41.16 (0.7)	34.2 (0.67)	
Unknown	6.6 (0.29)	6.26 (0.23)	7.34 (0.29)	6.21 (0.3)	
**Physical activity (% [SE])**					0.0001
low physical activity	28.38 (0.63)	28.3 (0.55)	27.78 (0.59)	29.17 (0.6)	
Moderate physical activity	24.82 (0.68)	23.41 (0.53)	24.98 (0.62)	26.42 (0.53)	
High physical activity	31.8 (0.75)	32.11 (0.56)	32.25 (0.73)	30.89 (0.62)	
Unknown	15 (0.63)	16.19 (0.49)	14.99 (0.42)	13.51 (0.53)	
BMI (kg/m²)	28.83 ± 0.10	23.24 ± 0.06	28.46 ± 0.04	36.36 ± 0.07	<0.0001
Diabetes (% [SE])	13.46 (0.46)	3.91 (0.21)	12.07 (0.37)	27.14 (0.6)	<0.0001
Hypertension (% [SE])	34.98 (0.76)	18.46 (0.46)	37.39 (0.64)	53.25 (0.64)	<0.0001
Sodium (mg/day)	3490.13 ± 21.53	3609.27 ± 37.18	3440.16 ± 18.18	3395.18 ± 19.63	<0.0001
Protein (g/day)	82.61 ± 0.51	85.89 ± 0.9	82.03 ± 0.39	79.11 ± 0.44	<0.0001
Serum creatinine (mg/dL)	0.89 ± 0.01	0.88 ± 0.02	0.9 ± 0.02	0.88 ± 0.02	<0.0001
Serum uric acid (mg/dL)	5.42 ± 0.02	5.01 ± 0.04	5.52 ± 0.02	5.83 ± 0.02	<0.0001
Total cholesterol (mg/dL)	194.98 ± 0.60	190.51 ± 0.92	200.07 ± 0.55	194.93 ± 0.55	<0.0001
ALT (Units/L)	25.39 ± 0.23	22.39 ± 0.3	26.5 ± 0.22	27.95 ± 0.25	<0.0001
AST (Units/L)	25.48 ± 0.19	24.83 ± 0.26	25.66 ± 0.18	26.09 ± 0.19	<0.0001
Triglycerides (mg/dL)	152.96 ± 1.91	115.52 ± 2.58	168.2 ± 1.79	183.32 ± 1.72	<0.0001
Albuminuria (% [SE])	9.15 (0.36)	6.27 (0.29)	8.21 (0.31)	13.87 (0.41)	<0.0001

GED, general educational development; PIR, poverty income ratio; BMI, body mass index; ALT, alanine transaminase; AST, aspartate transaminase.

**Table 2.** Correlation between body roundness index and albuminuria.

		OR¹ (95%CI²), *p*-value	
	Crude model (model 1)³	Minimally adjusted model (model 2)^4^	Fully adjusted model (model 3)^5^
**Continuous**	1.16 (1.14, 1.18) <0.0001	1.12 (1.10, 1.14) <0.0001	1.18 (1.12, 1.24) <0.0001
**Categories**			
**Tertile 1**	**Reference**	**Reference**	**Reference**
Tertile 2	1.34 (1.18, 1.52) <0.0001	1.02 (0.90, 1.16) 0.7382	0.91 (0.79, 1.05) 0.2128
Tertile 3	2.41 (2.13, 2.73) <0.0001	1.73 (1.52, 1.96) <0.0001	1.24 (1.02, 1.51) 0.0333
P for trend	<0.0001	<0.0001	0.0421

In insensitivity analysis, the body roundness index was converted from a continuous variable to a categorical variable (tertiles).

^1^ OR: Odds ratio.

^2^ 95% CI: 95% confidence interval.

^3^ Model 1: No covariates were adjusted.

^4^ Model 2: Adjusted for sex, age, and race.

^5^ Model 3: Adjusted for sex, age, race, education level, smoking status, drinking status, poverty income ratio, physical activity, sodium intake, protein intake, ALT, AST, total cholesterol, serum creatinine, triglycerides, serum uric acid, BMI, hypertension, diabetes.

**Table 3.** Threshold effect analysis of BRI on albuminuria.

	Total sample	Male	Female
**Fitting by standard liner model**			
OR^1^ (95%CI^2^)	1.18 (1.12, 1.24)	1.31 (1.24, 1.39)	1.11 (1.05, 1.18)
*P*-value	<0.0001	<0.0001	<0.0001
**Fitting by two-piecewise linear model**			
Breakpoint (K)	3.08	3.15	2.91
OR1 (<K)	0.61 (0.44, 0.84)	0.52 (0.30, 0.89)	0.57 (0.34, 0.96)
	0.0027	0.0200	0.0361
OR2 (≥K)	1.19 (1.13, 1.25)	1.21 (1.09, 1.33)	1.15 (1.08, 1.23)
	<0.0001	0.004	<0.0001
Logarithmic likelihood ratio test *p*-value	<0.001	<0.001	<0.001

^1^ OR: Odds ratio.

^2^ 95% CI: 95% confidence interval.

### The correlation between BRI and albuminuria

Across all logistic regression models, BRI demonstrated a positive correlation with albuminuria risk. In the unadjusted model, each 1-unit increment in BRI was associated with a 16% higher likelihood of albuminuria (OR: 1.16, 95% CI: 1.14–1.18). This association persisted after minimal adjustment for sex, age, and race (OR: 1.12, 95% CI: 1.10–1.14) and was strongest in the fully adjusted model, which controlled for a comprehensive set of covariates (OR: 1.18, 95% CI: 1.12–1.24). When BRI was analyzed as a categorical variable, participants in Tertile 3 had a significantly greater risk of albuminuria in comparison with Tertile 1. In the fully adjusted model, the probability of albuminuria was 24% higher in Tertile 3 (OR: 1.24, 95% CI: 1.02–1.51), with a significant trend observed across tertiles (p for trend = 0.0421) (see Table 2). Results from multiple imputation analysis were comparable to the complete-case findings, confirming the robustness of the observed association between BRI and albuminuria (Supplementary S1).

To evaluate whether BRI provided additional predictive value beyond conventional obesity metrics, we compared its association with albuminuria against that of BMI. Substituting BRI with BMI in the fully adjusted model yielded a weaker association (OR: 1.09, 95% CI: 1.06–1.12; *p* < 0.0001) compared to the model using BRI (OR: 1.18, 95% CI: 1.12–1.24). In the joint model, BRI remained significantly associated with albuminuria (OR: 1.14, 95% CI: 1.07–1.21; *p* < 0.001), whereas BMI was not (OR: 1.01, 95% CI: 0.98–1.04; *p* = 0.21). These results suggest that BRI may better capture the risk of albuminuria than BMI. Full model comparisons are shown in Supplementary S2.

**Figure 2.**
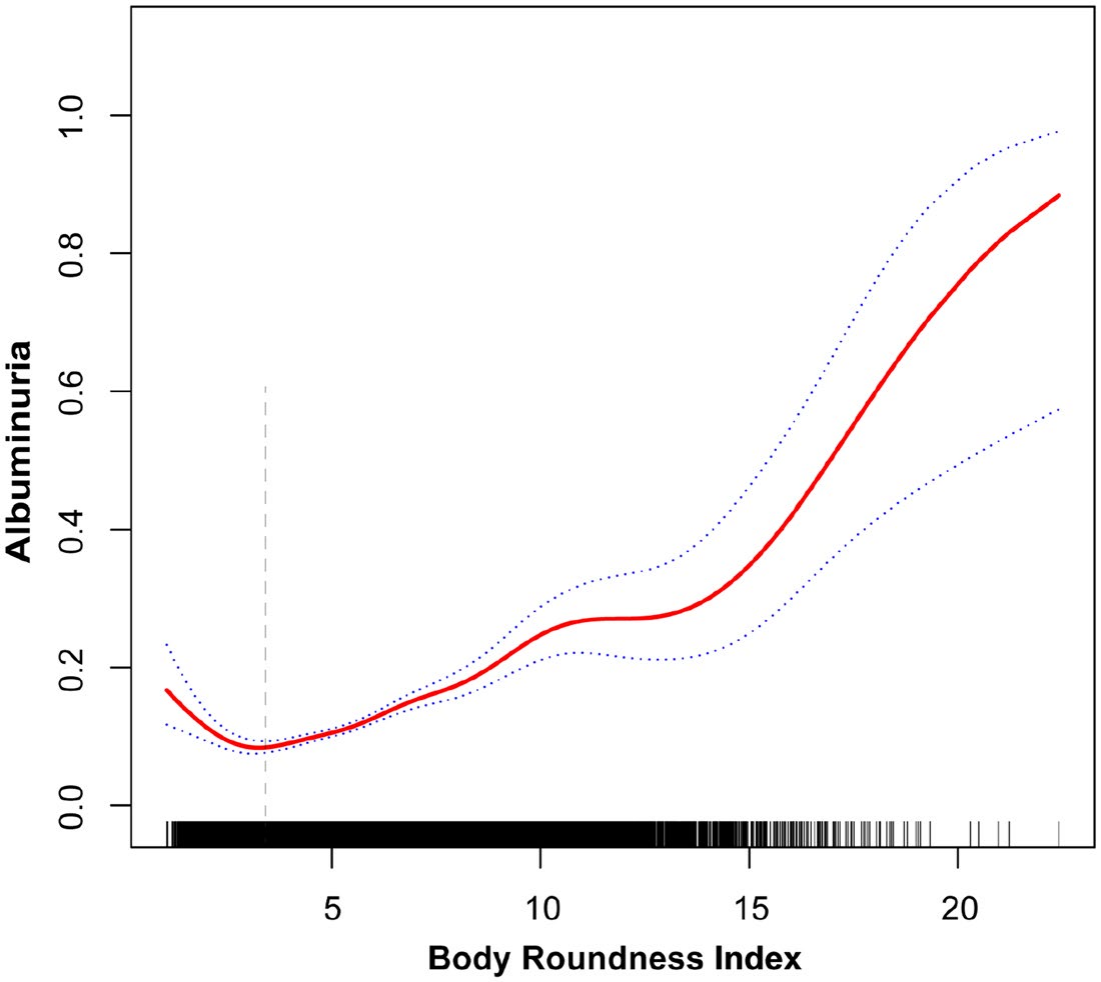
**Smooth curve fitting for BRI and albuminuria.** A non-linear J-shaped relationship between BRI and albuminuria was detected. A vertical dashed line indicates the estimated inflection point at BRI = 3.08.

Further analysis identified a non-linear J-shaped correlation, with an inflection point at a BRI value of 3.08 (Log-likelihood *p* < 0.0001) (Figure 2). Below this threshold, BRI exhibited a significant negative association with albuminuria, as indicated by an odds ratio of 0.59 (95% CI: 0.43–0.83). In contrast, beyond the inflection point, the association became significantly positive, with an odds ratio of 1.12 (95% CI: 1.15 to 1.28, *p* < 0.0001) (Table 3).

### Subgroup analysis

**Figure 3.**
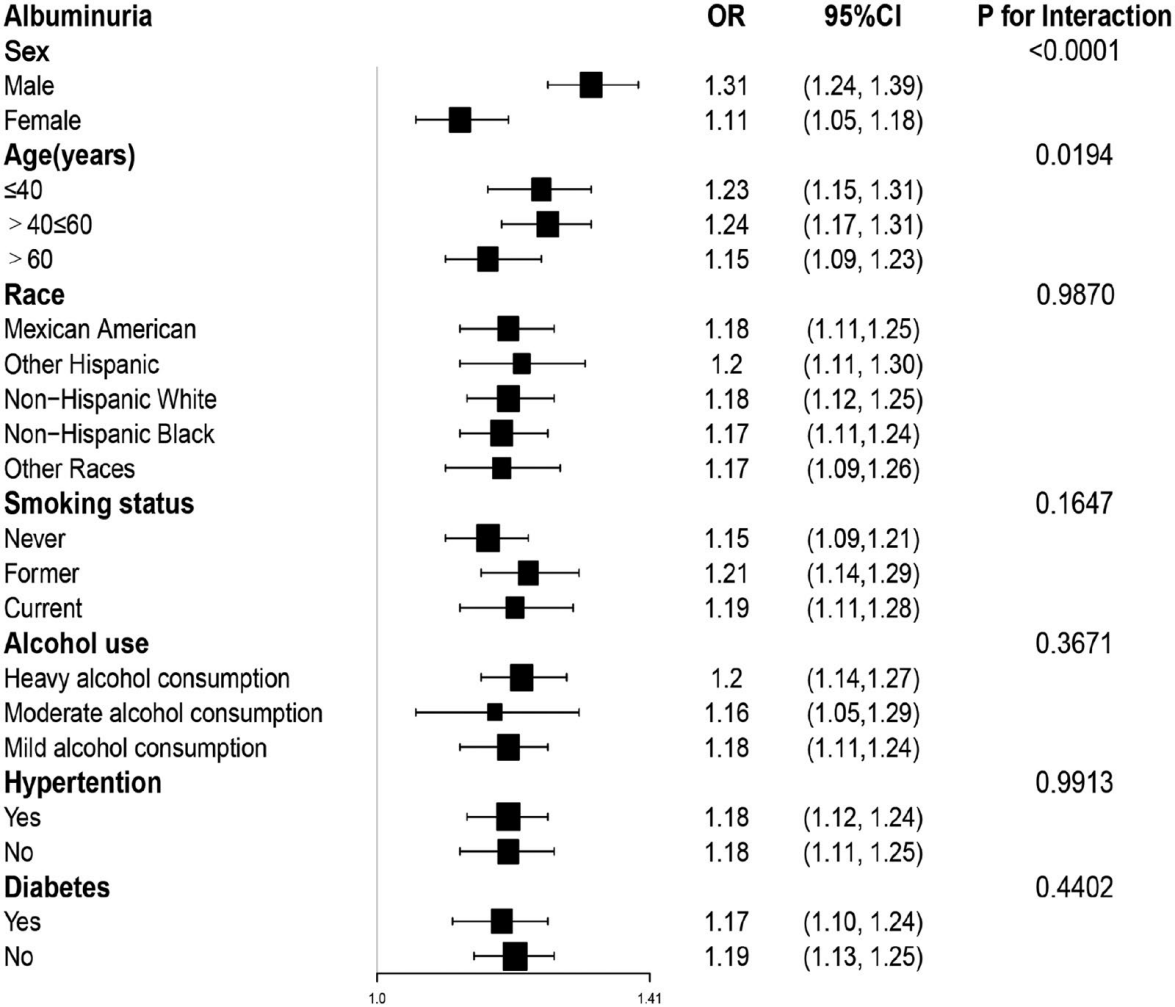
**Subgroup analysis for the association between BRI and albuminuria.** Subgroup analysis revealed a consistent relationship between BRI and albuminuria across various factors, with sex showing the strongest interaction effect.

Subgroup investigations showed that the relationship between BRI and albuminuria remained consistent across most factors, including race, hypertension, and diabetes, with interaction p-values greater than 0.05. However, significant effect modification was observed for both sex (P for interaction < 0.0001) and age (*p* = 0.0194), as confirmed by formal interaction testing. Specifically, the association was stronger in males (OR: 1.31, 95% CI: 1.24–1.39) compared to females (OR: 1.11, 95% CI: 1.05–1.18). These findings are illustrated in Figure 3, which also shows that higher BRI was generally associated with increased odds of albuminuria in all subgroups (Figure 3).

**Figure 4.**
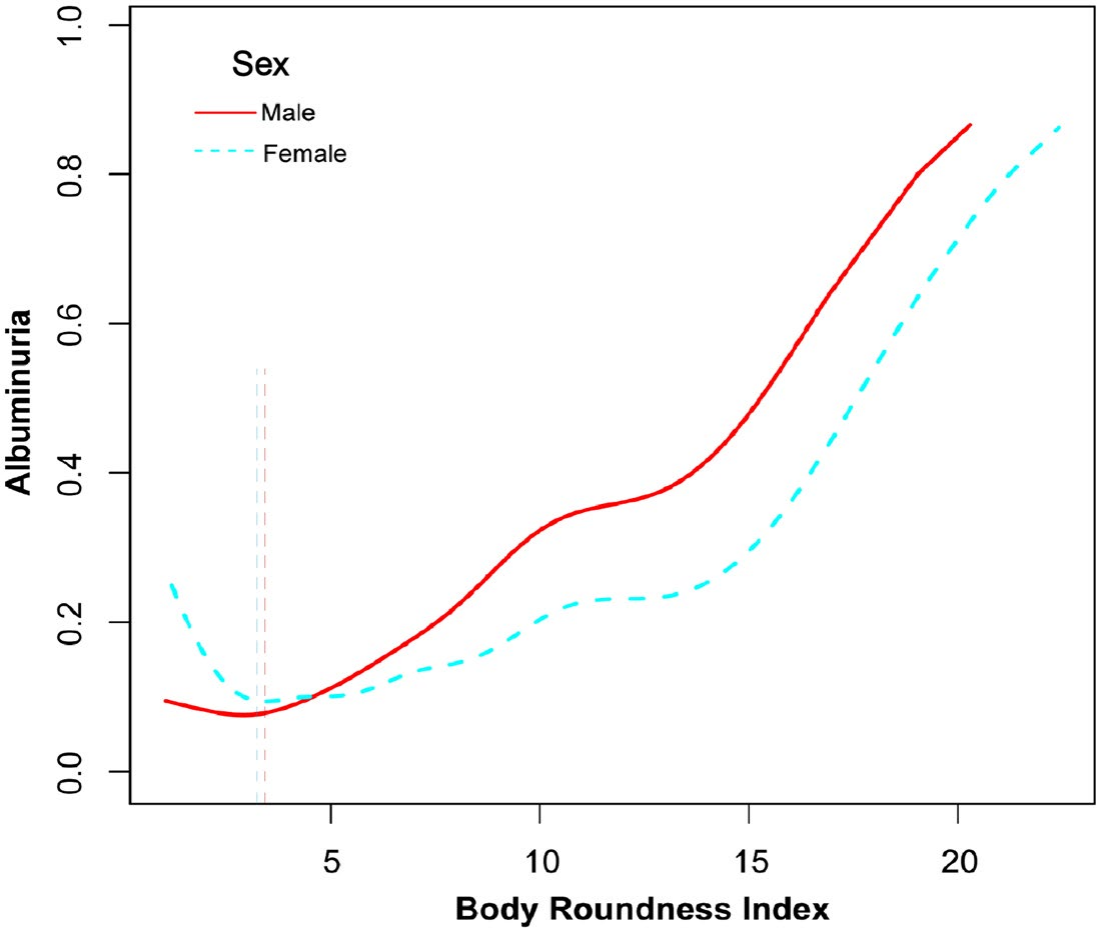
**Relationship between BRI and albuminuria by sex.** Sex-stratified dose-response analysis showed a J-shaped curve between BRI and albuminuria, with inflection points at BRI 3.15 for males and 2.91 for females. Below these thresholds, a negative association was observed, while above them, the relationship became positive, highlighting significant sex-specific variations. Vertical dashed lines indicate the sex-specific inflection points.

Further investigation into the dose-response relationship revealed a J-shaped curve linking BRI to albuminuria in both sex subgroups (Figure 4). In males, the inflection point was identified at a BRI value of 3.15 (Log-likelihood *p* < 0.0001). Below this value, a significant inverse relationship between BRI and albuminuria was identified (OR: 0.52, 95% CI: 0.30–0.89; *p* = 0.0200), whereas above 3.15, the relationship turned positive (OR: 1.21, 95% CI: 1.09–1.33; *p* = 0.004). In females, the breakpoint occurred at a BRI value of 2.91 (Log-likelihood *p* < 0.0001), with a no negative association before this threshold (OR: 0.57, 95% CI: 0.34–0.96; *p* = 0.0361) and a positive association afterward (OR: 1.15, 95% CI: 1.08–1.23; *p* < 0.0001) (Table 3).

Sex, age, race, education level, smoking status, drinking status, poverty income ratio, physical activity, sodium intake, protein intake, ALT, AST, total cholesterol, serum creatinine, triglycerides, serum uric acid, BMI, hypertension, diabetes were adjusted.

## Discussion

This study identified a positive association between BRI and albuminuria. Further analysis revealed a non-linear J-shaped relationship, with a breakpoint at a BRI value of 3.08. Subgroup analysis showed that the correlation between BRI and albuminuria was more pronounced in males in comparison with females. Specifically, in the male subgroup, the J-shaped curve exhibited an inflection point at a BRI value of 3.15, while in females, the inflection point was found at a BRI value of 2.91.

BRI is a newly proposed obesity indicator that, in contrast to the conventional body mass index (BMI), more accurately reflects body fat distribution, particularly central obesity (abdominal obesity) [12]. This distinction is crucial as abdominal obesity has been shown to be more strongly correlated with albuminuria [19]. Several studies have established the connection between abdominal obesity and albuminuria. For example, Wen-yuan Lin et al. found a higher incidence of albuminuria in Chinese individuals with central obesity [20]. Similarly, Ga Eun Nam et al. reported a no relationship between central obesity and elevated albuminuria incidence in Korean women [21]. A recent study also found that visceral fat accumulation, which is closely associated with BRI, was positively linked to increased urine albumin excretion in patients with type 2 diabetes [22]. Another study also found that those with abdominal obesity (measured by A Body Shape Index) could be considered high-risk individuals with albuminuria [23]. In line with previous findings, our research also confirmed that BRI was positively correlated with the incidence of albuminuria after the inflection point (BRI = 3.08). Albuminuria typically results from impaired renal filtration, which involves damage to the glomerular filtration membrane, alterations in the charge barrier, podocyte destruction, and elevated filtration pressure [24]. These mechanisms are often aggravated by abdominal obesity, a condition that BRI effectively identifies. However, it should be noted that BRI is an indirect anthropometric measure and not a direct assessment of body fat or visceral adiposity. While it correlates with central obesity, its estimation is based on waist circumference and height rather than imaging-based or biochemical quantification of adipose tissue. Numerous studies have explained the pathophysiological mechanisms by which obesity affects glomerular filtration. First, Kotaro Haruhara et al. found that podocyte apoptosis plays a critical role in obesity-related glomerular lesions, a process that is more prominent in those with central obesity, which BRI effectively measures [25]. Second, in obese individuals, the renin-angiotensin-aldosterone system is upregulated, leading to increased glomerular filtration pressure [26]. Finally, adipocytes release various pro-inflammatory cytokines such as IL-6, TNF-α, IL-1, and MCP-1, contributing to oxidative stress and damage to the glomerular filtration membrane, all of which are more prevalent in individuals with abdominal obesity [27,28].

In addition, the present study observed an inverse relationship between BRI and albuminuria risk prior to the inflection point (BRI = 3.08), consistent with findings from previous research. Cheol Min Jang et al. reported that underweight participants, likely corresponding to individuals with extremely low BRI, exhibited a significantly higher risk of albuminuria as opposed to those within the normal weight range [29]. This suggests that deviations from an optimal BRI range, in either direction, might elevate urinary albumin levels. The increased albuminuria risk in individuals with low BRI might be attributed to malnutrition, which can lead to systemic changes such as compromised vascular integrity or immune dysfunction, ultimately affecting renal health [30]. While direct epidemiological evidence supporting the increased risk of albuminuria at low BRI levels is limited, biologically plausible mechanisms include undernutrition-related endothelial dysfunction, loss of lean body mass, and reduced anti-inflammatory adipokine secretion. These hypotheses require further validation in future studies.

In our comparative analysis, BRI demonstrated a stronger association with albuminuria than BMI. When substituted into the fully adjusted model, BMI remained significantly associated with albuminuria, but with a smaller effect size than BRI. Moreover, when BRI and BMI were included in the same model, BRI retained its statistical significance while BMI did not, suggesting that BRI provides additional and possibly superior predictive value for albuminuria beyond general adiposity.

The clinical advantage of BRI may lie in its ability to capture visceral adiposity more accurately. Unlike BMI, which reflects overall body mass and does not distinguish between fat and lean mass, BRI incorporates waist circumference and height to better estimate abdominal fat accumulation. Visceral fat has been shown to be more metabolically active and more strongly associated with insulin resistance, systemic inflammation, and endothelial dysfunction—mechanisms that are directly implicated in the development of albuminuria. Therefore, BRI may reflect pathogenic processes that BMI fails to detect, offering a more precise tool for identifying individuals at risk of early renal damage.

The subgroup analysis revealed sex differences. Compared to females, males showed significantly stronger associations between BRI and albuminuria. This observation was supported by MC Foster et al.’s study, which found that males had a more significant correlation between visceral adipose tissue and microalbuminuria as compared to females [31]. Biological differences, particularly variations in sex hormones, are likely contributing factors. While males may exhibit heightened susceptibility, estrogen appears to play a protective role in females. For instance, an animal study by Xiaoxin X Wang et al. showed that estrogen-related receptor agonists reduced urinary albumin excretion [32], and a multiracial cohort study by Andrea G Kattah et al. found that estrogen therapy was linked to a decreased incidence of albuminuria in postmenopausal women [33]. Both animal and human experimental studies demonstrated that estrogen might have a renoprotective effect, thereby reducing the occurrence of albuminuria. Moreover, the identification of J-shaped relationships between BRI and albuminuria, with distinct inflection points for males (BRI = 3.15) and females (BRI = 2.91), highlights the importance of developing sex-specific BRI reference ranges. Such tailored thresholds could improve risk assessment and guide clinical interventions more effectively. Future research should not only explore the mechanisms underlying these sex differences but also verify these thresholds in diverse populations.

This study has several no strengths. First, this study collected data from NHANES, a large, nationally representative database of the U.S. population, which enhances the representativeness and reliability of the findings. Second, the study involved a large sample size, ensuring sufficient statistical power to detect relationships and perform subgroup analyses across diverse demographic and health subgroups. Third, NHANES data includes standardized and high-quality measurements, ensuring the accuracy and consistency of key variables such as the Body Roundness Index and albuminuria. This study also has several limitations. First, even though it considered a variety of covariates, many confounders were still difficult to identify and control. Second, causality cannot be confirmed in cross-sectional studies. Third, although we adjusted for hypertension and diabetes in all regression models, participants in the highest BRI tertile had a higher prevalence of these conditions. Therefore, we cannot fully exclude the possibility that the observed association between BRI and albuminuria may, in part, be mediated or confounded by hypertension and/or diabetes. Fourth, our findings are based solely on NHANES data and may not be generalizable to other populations. Furthermore, very few participants in this dataset were taking SGLT2 inhibitors or GLP-1 receptor agonists, which are now known to reduce albuminuria. The lack of data on these medications may limit the applicability of our results to contemporary clinical settings.

## Conclusion

In conclusion, a significant J-shaped relationship was observed between BRI and albuminuria, with a breakpoint at a BRI value of 3.08. The findings suggest that maintaining an optimal BRI could play a crucial role in preventing albuminuria. However, additional longitudinal studies are needed to confirm causality and provide more solid insights into the underlying mechanisms, which would inform the development of effective preventive strategies.

## Supplementary Material

Table S1.docx

Table S2.docx

## Data Availability

This study is based on publicly available data from the National Health and Nutrition Examination Survey (NHANES), conducted by the National Center for Health Statistics (NCHS). The NHANES datasets from 2005 to 2020 are freely accessible at https://www.cdc.gov/nchs/nhanes/index.htm. All data used in this study were obtained following the NHANES data usage guidelines, and no additional data were generated or analyzed beyond the publicly available datasets.
