# Supplementary material for: A J-shaped link between body roundness index and albuminuria in U.S. adults: insights from NHANES 2005–2020
Source: Ren Fail. 2025 Oct 8;47(1):2567035. doi: 10.1080/0886022X.2025.2567035 (PMC12509294; doi:10.1080/0886022X.2025.2567035)
Supplement: Table S1.docx [file IRNF_A_2567035_SM7889.docx]

**TABLE S1**

**Comparison of Complete-Case Analysis vs. Multiple Imputation Analysis for the Association between BRI and Albuminuria**

| **Analysis Type** | **OR(95% Cl)** | **P-value** |
| --- | --- | --- |
| Complete-case analysis | 1.18 (1.12–1.24) | <0.0001 |
| Multiple imputation (MICE) | 1.17 (1.11–1.23) | <0.0001 |
