# Supplementary material for: A J-shaped link between body roundness index and albuminuria in U.S. adults: insights from NHANES 2005–2020
Source: Ren Fail. 2025 Oct 8;47(1):2567035. doi: 10.1080/0886022X.2025.2567035 (PMC12509294; doi:10.1080/0886022X.2025.2567035)
Supplement: Table S2.docx [file IRNF_A_2567035_SM7888.docx]

**TABLE S2**

**Comparison of Multivariable Logistic Regression Models for the Association Between Anthropometric Indicators (BRI and BMI) and Albuminuria**

| **Model** | **Variables included** | **OR(95% Cl)** | **P-value** |
| --- | --- | --- | --- |
| A | BRI Only | 1.18 (1.12–1.24) | <0.0001 |
| B | BMI Only | 1.09 (1.06–1.12) | <0.0001 |
| C | BRI + BMI | BRI: 1.14 (1.07–1.21)  BMI: 1.01 (0.98–1.04) | <0.0001  BMI: 0.21 |
